# THE MOLECULAR CANCER SUBTYPES VERSUS THE INDUSTRY ARSENAL. WHICH ONE DRIVES GASTRIC CANCER TREATMENT?

**DOI:** 10.1590/0102-6720202400018e1811

**Published:** 2024-07-01

**Authors:** Paulo Pimentel de ASSUMPÇÃO, Paulo KASSAB

**Affiliations:** 1 Universidade Federal do Pará, Oncology Research Center – Belém (PA), Brazil;; 2 Santa Casa de São Paulo, Faculty of Medicine, Department of Surgery – São Paulo (SP), Brazil.

**Keywords:** Stomach Neoplasms, Molecular Medicine, Proteomics, Genomics, Oncology, Neoplasias Gástricas, Medicina Molecular, Proteômica, Genômica, Oncologia

## Abstract

Molecular medicine opened new horizons in understanding disease mechanisms and discovering target interventions. The wider availability of DNA and RNA sequencing, immunohistochemical analysis, proteomics, and other molecular tests changed how physicians manage diseases. The gastric cancer molecular classification proposed by The Cancer Genome Atlas Program divides gastric adenocarcinomas into four subtypes. However, the available targets and/or immunotherapies approved for clinical use seem to be dissociated from these molecular subtypes. Until a more reliable interpretation of the stupendous amount of data provided by the molecular classifications is presented, the clinical guidelines will rely on available actionable targets and approved therapies to guide clinicians in conducting cancer management in the era of molecular therapies.

## RESUMO

A medicina molecular abriu novos horizontes na compreensão dos mecanismos das doenças e na descoberta de intervenções alvo. A maior disponibilidade de sequenciação de DNA e RNA, análise imuno-histoquímica, proteômica e outros testes moleculares mudou a forma como os médicos conduzem as doenças. A classificação molecular do câncer gástrico proposta pelo Atlas do Genoma do Câncer divide os adenocarcinomas gástricos em quatro subtipos. No entanto, os alvos disponíveis e/ou imunoterapias aprovadas para uso clínico parecem estar dissociados desses subtipos moleculares. Até que seja apresentada uma interpretação mais confiável da estupenda quantidade de dados fornecidos pelas classificações moleculares, as diretrizes clínicas irão se basear nos alvos acionáveis disponíveis e nas terapias aprovadas para orientar os médicos na condução da gestão do câncer na era das terapias moleculares.

## INTRODUCTION

Molecular medicine opened new horizons in understanding disease mechanisms and discovering target interventions. The wide availability of DNA and RNA sequencing, immunohistochemical analysis, proteomics, and other molecular tests changed how physicians manage diseases.

This revolution reached almost every field of medicine but was especially incorporated into oncology. Cancers that were unresponsive to classical therapies are now treated with great success, as a consequence of both understanding their molecular mechanisms and the development of specific target therapies to hone in on them.

Academic research institutions and the pharmaceutical industry have triggered the discovery of many new sensitive targets, translating them into daily practice.

This development in molecular understanding gave rise to the precision medicine initiative, which emerged as a strategy to treat patients more efficiently, according to both the molecular features of the tumor and the peculiarities of the individual genetic background, including pharmacogenetic variants.

Diverse initiatives to classify cancers beyond the traditional morphological histological criteria, which incorporate the molecular patterns of the tumors and aim to guide their clinical management, have emerged and become extensively reported in medical journals^1,6^.

Nevertheless, in the real world, treatment decisions are mostly driven by available target therapies based on protocols of potential clinical response, instead of by molecular classifications^10^.

The Cancer Genome Atlas Program (TCGA) consortium provided molecular classifications of many cancer types, including gastric, colonic, and others. The scientific community expected to translate these classifications into clinical decisions, but this goal seems very far from the daily practice^5^.

The gastric cancer molecular classification proposed by the TCGA divides gastric adenocarcinomas into four subtypes. However, the available targets and/or immunotherapies approved for clinical use seem to be dissociated from these molecular subtypes^5^.

Anti-HER-2 therapies, although most commonly applied to the Chromosomal instability molecular subtype (CIN), are also indicated for tumors from the other subtypes, as long as they present high human epidermal growth factor receptor-type 2 (HER-2) expression. The same is true for Claudin 18-2. Target therapy will be available for every molecular subtype according to antibody reactivity to Claudin-2. Anti-PD-1 therapy is indicated for a specific molecular subtype, the Microsatellites Instability molecular subtype (MSI). But even for this indication, if a tumor from other molecular subtypes presents a high expression of PD-1 (combined positive score [CPS]>1), this therapy will be made available for clinical use. Moreover, Epstein-Barr virus (EBV) subtypes and cancers with a high tumor mutation burden also represent potential indications for such therapy^3,4^.

These “directed by available therapy” classifications are also present in the management of many other cancer types, such as lung, breast, and melanoma, among others.

The agnostic indication is the most extreme example of treatment guided by available actionable target therapies. Accordingly, if the target is presented, treatment will be indicated, regardless of the tumor type or site of origin, whether it is gastric, colonic, pulmonary, uterine, or other cancers; once the target is present, a drug will be indicated. Should this still be called “precision medicine”?^1,7^.

The dissociation between the molecular classification and indications for specific therapies does not diminish the importance of the molecular classifications. These classifications provide robust information for the development of new therapies and shed light on the understanding of each cancer molecular mechanism, allowing future interventions in treatment, diagnosis, and prevention strategies, the latter being the most effective future cancer control^8,9^.

Until a more reliable interpretation of the stupendous amount of data provided by the molecular classifications is presented, the clinical guidelines will rely on available actionable targets and approved therapies to guide clinicians in conducting cancer management in the era of molecular therapies. Currently, the available industry arsenal has greater weight than the molecular classifications themselves in the clinical treatment decision-making^2,3^.
